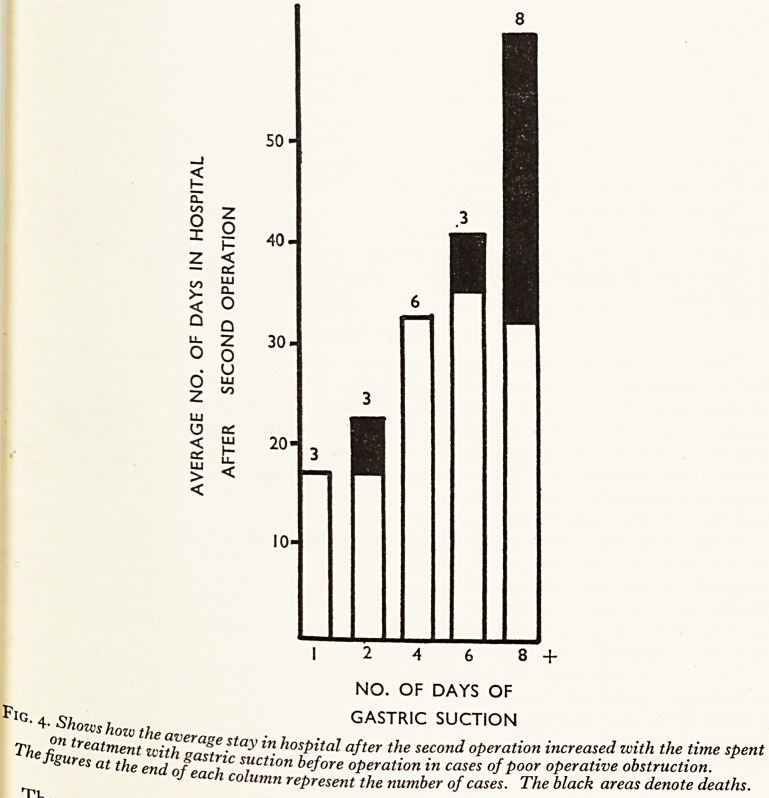# A Review of Acute Intestinal Obstruction

**Published:** 1959-04

**Authors:** P. J. W. Monks

**Affiliations:** The Royal Infirmary, Bristol


### A REVIEW OF ACUTE INTESTINAL OBSTRUCTION

BY

P. J. W. MONKS

*The Royal Infirmary, Bristol*

Acute intestinal obstruction is a common and serious surgical emergency. Vick 1932) reported a mortality of 26 per cent in a collected series of nearly 7,000 cases, ncluding 322 cases from Bristol Royal Infirmary. This high mortality has been educed in this series to 16 per cent, but this still seems a high figure although it is in keeping with a recently reported series by Smith, Perry and Yonehiro (1953), and is ilso the figure quoted in *British Surgical Practice* (1948). This high mortality could indoubtedly be reduced by earlier diagnosis.

Between January 1952 and June 1957, 5,131 emergency operations were carried out n the general surgical department of the Bristol Royal Infirmary; 440 of these operaions were for acute intestinal obstruction and these cases are now reviewed. Table 1 hows the main causes of obstruction and sets out the number of cases in each group ind their respective mortality. For comparison, Vick's figures of 1932 are shown where these are comparable.

TABLE 1

Acute intestinal obstruction: showing the main causes in 440 cases, and the mortality in each group. The mortality is compared with the larger series published by Vick, 25 years ago.

| Cause of Obstruction | Cases | Deaths | % Mortality | % Mortality Vick 1932 |
|---|---|---|---|---|
| Femoral hernia .. .. .. | 104 | 6 | 6 | 15·9 |
| Inguinal hernia .. .. .. | 99 | 10 | 10 | 11·5 |
| Ventral hernia .. .. .. | 27 | 7 | 25 | 38 |
| Total external hernia .. .. | 230 | 23 | 10 | — |
| Obstruction with no mention of strangulation .. .. .. | 65 | 13 | 20 | 32·9 |
| Obstruction with strangulation .. | 67 | 2 | 3 | 34 |
| Colon obstruction .. .. .. | 45 | 21 | 47 | — |
| Post-operative .. .. .. | 25 | 6 | 25 | — |
| Vascular .. .. .. .. | 8 | 6 | 70 | — |
| Total .. .. .. .. | 440 | 71 | 15·5 | 26·2 |

### EXTERNAL HERNIA

Strangulated external hernia was the commonest cause of obstruction and occurred in 230 cases, just over half of the total, and there were 23 deaths (10 per cent). The site of the hernia had a very direct bearing on the mortality, and important differences in each group of herniae were apparent.

Strangulated femoral hernia occurred 104 times, and resulted in 6 deaths (6 per cent), strangulated inguinal hernia 99 times causing 10 deaths (10 per cent). Femoral hernia presented at a later age than inguinal hernia (see Fig. 1), the youn case was 45 and the highest incidence was in the 70–79 age group; inguinal he were found at all ages and the peak incidence was in the 60–69 age group. Age see

FIG. 1. *Compares the age incidence in cases of strangulated inguinal and femoral herniae. The areas represent deaths.*

to have little effect *per se* on the mortality of strangulated femoral hernia for no 16 cases over the age of 80 died, and only 4 of the 55 cases over the age of 70 In the inguinal hernia group, however, age was an important factor for there w deaths in 33 cases over the age of 70.

All the deaths from strangulated femoral hernia occurred in late cases, all desperately ill on admission and in spite of vigorous intravenous fluid replacem the deaths occurred within 24 hours of operation, and all were due to peripheral circulatory failure. By comparison the deaths from strangulated inguinal hernia occurred in both early and late cases (see Fig. 2), and the cause of death was different:

FIG. 2. *Compares the length of history in cases of strangulated inguinal and femoral herniae. The black areas represent deaths.*

only 1 patient died of peripheral circulatory failure shortly after operation, (this was the youngest death, a man of 45 who had been vomiting severely for 5 days, and was in gross electrolyte imbalance on admission); the other deaths were all in the elderly and were mainly due to associated cardio-respiratory complications, and occurred later in the post-operative period: one patient died of heart failure, 1 of a cerebro-vascular accident and 3 of pulmonary embolus; the other deaths were more directly due to the obstruction, for 1 inhaled vomit whilst still in the casualty department, 1 old man of 90 had total gangrene of the whole small bowel, and 1 died of a leaking suture line following a resection.

Resection was required in 25 cases of strangulated femoral hernia. Twelve of th[illegible] had been strangulated for over 3 days, one for 26 days, and 3 of these died; the oth[illegible] with a shorter history all survived. Three cases requiring a resection had a histor[illegible] less than 12 hours; one of these was a Maydl's hernia with the strangulated loop ly[illegible] within the abdomen. Only 9 cases of inguinal hernia needed a resection and 2 di[illegible] There were 2 cases of reduction-en-masse of an inguinal hernia, the patient in b[illegible] cases reducing the hernia himself.

Femoral hernia seems to carry a lower mortality than strangulated inguinal he[illegible] in spite of the older average age, higher resection rate, and longer average history. T[illegible] reason for this would seem to lie in the difficulty of reducing and repairing an o[illegible] large inguinal hernia in an elderly patient with associated cardio-respiratory dise[illegible] These patients had often been refused elective surgery for their hernia because of th[illegible] poor general condition. In comparison the femoral hernia is usually small and [illegible] relatively easy to relieve the strangulation. About half these cases were approach[illegible] from below the inguinal ligament, thus no muscles were cut and an easy convalesce[illegible] ensured.

Strangulated ventral and umbilical hernia had a very high mortality, for there w[illegible] 7 deaths in 27 cases (25 per cent). Five cases required resection and three of th[illegible] died. This very high mortality rate was in part due to the attempted repair of v[illegible] large herniae, for 2 cases died within 24 hours of operation with gross respirat[illegible] insufficiency, and 1 case died of a burst abdomen on the seventh post-operative d[illegible] These patients were often very obese and several were diabetic; these factors w[illegible] important contributory causes of death.

OBSTRUCTION WITHOUT MENTION OF STRANGULATION

There were 65 cases of small bowel obstruction without strangulation, and 13 ca[illegible] died (20 per cent). The commonest cause of obstruction in this group was adhesion[illegible] there were 40 such cases. The adhesions were all multiple and often very extens[illegible] most followed previous operations. Other causes were metastases, 7 cases; appen[illegible] citis (either retro-ileal or with a pelvic abscess), 6 cases; gall stone ileus, 4 cases; sim[illegible] stricture, 2 cases; carcinoma, 3 cases; simple tumour, Crohn's disease, Mecke[illegible] diverticulum, 1 case each. Two of the deaths were due to multiple metastas[illegible] Appendicitis, gall-stone ileus and carcinoma of the jejunum caused 1 death each. T[illegible] remaining 8 deaths were all due to multiple adhesions.

These cases of so-called "simple" obstruction frequently had an insidious ons[illegible] often with previous minor attacks of colic and vomiting. Many had several days histo[illegible] by the time they reached hospital (see Fig. 3). Only 9 cases had a history of less th[illegible] 24 hours and a third had a history longer than 5 days. As a result many were ill a[illegible] dehydrated—sometimes severely so—by the time they reached hospital, and treatme[illegible] for several hours with intravenous fluids was necessary before they were consider[illegible] fit for surgery.

The diagnosis of established intestinal obstruction was fairly obvious by the ti[illegible] these patients reached hospital, although the cause of the obstruction was not alwa[illegible] clear. Many of these cases had plain supine and erect X-rays of the abdomen taken [illegible] admission, these showed various degrees of intestinal dilatation associated with flu[illegible] levels and confirmed the diagnosis of obstruction in nearly every case. However the[illegible] were of little value in demonstrating the cause of obstruction or the presence [illegible] strangulation.

Difficult operations to relieve these obstructions were sometimes necessary, especiall[illegible] in those cases with extensive adhesions. Two cases had such dense adhesions that [illegible] was considered only possible to do an enterostomy between dilated and collapsed bowe[illegible] Three cases developed further obstruction post-operatively and required a secon[illegible] operation. The lateness of these cases, together with the sometimes formidabl[illegible] operations in the presence of gross intestinal dilatation, account for the high mortality[illegible]

A few of these cases were treated by gastric suction and intravenous fluids for several days; this led to further delay and the impression is that these cases did not do so well as those operated on as soon as their fluid balance had been restored.

FIG. 3. *Compares the length of history in cases of obstruction with and without strangulation. The black areas represent deaths.*

OBSTRUCTION WITH STRANGULATION

This group had the lowest mortality of all, there were 65 cases and 7 required re-section, and there were only 2 deaths (3 per cent). One patient died of congestive cardiac failure on the seventh post-operative day, and the other case was misdiagnosed as a dissecting aneurysm and the strangulation was only found post-mortem. The commonest cause of strangulation was a band, often single, this was found 46 times.

Other causes were—internal hernia 9 cases, intussusception 9 cases, volvulus o[illegible] small gut 2 cases, Meckel's diverticulum 1 case.

The initial severe pain brought these patients to hospital much earlier than t[illegible] without strangulation. Fig. 3 illustrates this point; the great majority of cases [illegible] admitted within 24 hours and many within a few hours of the onset of the pai[illegible] nine cases there was considerable delay in diagnosis after the patient was se[illegible] hospital, the most common provisional diagnosis was renal colic. One case w[illegible] diagnosed for five days before laparotomy showed the true state of affairs, and an[illegible] thought to be a dissecting aneurysm until post-mortem examination showed a lo[illegible] small bowel strangulated by a simple band. The severe pain is commonly referr[illegible] the back, probably due to involvement of the mesentery, and it is all too eas[illegible] interpret this as renal pain. Cases with a tight strangulation may only vomit once i[illegible] early stages and often the bowel acts after the onset of the pain. The early phy[illegible] signs are minimal, there is no distension, little rigidity, and sometimes very [illegible] tenderness. This lack of physical signs in the abdomen together with a negative X[illegible] examination lead to the difficulty in diagnosis when these cases are first seen.

Plain X-rays of the abdomen were taken in 30 of these cases. No evidence o[illegible] struction was reported in 6, paralytic ileus in 2, subacute or partial obstruction [illegible] and complete obstruction was only reported in 13 cases or just under half. The neg[illegible] findings were all in early cases, for in these the small bowel has not had time to dis[illegible] and the colon and rectum still frequently contain gas. Whilst fluid levels and di[illegible] loops are characteristic of a small gut obstruction that has been present for 24 ho[illegible] a relative absence of gas is often the most important sign of an early strangula[illegible] Plate XVIII shows a plain film of the abdomen 2 hours after the onset of pain, the [illegible] an almost complete absence of gas in the small bowel, gas is present in the colon and t[illegible] is a suggestion of a soft tissue mass. At laparotomy several feet of small bowel [illegible] found tightly snared by a band, the bowel above the obstruction being quite emp[illegible]

Major small bowel strangulation rapidly produces shock, from loss of blood [illegible] plasma into the bowel lumen, the bowel wall, and the peritoneum. In the later cases [illegible] must be replaced as rapidly as possible prior to operation. Occasionally cases are [illegible] when the shock develops very early, is often profound within an hour or two, an[illegible] sponds poorly to adequate blood and plasma transfusions, until the strangulatio[illegible] relieved. In these cases it is best to operate almost as soon as an efficient plasma [illegible] is running for dramatic improvement was particularly well seen in the two extre[illegible] ill cases of complete small bowel volvulus, where the blood pressure returned to no[illegible] as soon as the volvulus was untwisted.

POST-OPERATIVE OBSTRUCTION

There were 25 cases of intestinal obstruction developing after operation, wh[illegible] the patient was still in hospital. This is a serious complication for 6 (25 per cent[illegible] these patients died. These cases are divided into 2 groups, the first arising withi[illegible] hours of the primary operation, the second after a period of well being, usually ab[illegible] the seventh post-operative day.

Some of these cases were due to internal hernia or volvulus, and they had se[illegible] abdominal pain. Others were the result of organizing adhesions and followed a pe[illegible] of post-operative ileus, one condition gradually merging into the other. It was in th[illegible] latter cases that most difficulty was experienced in deciding the right time to re-oper[illegible]

In the early post-operative group there were 8 cases, two of these were not recogni[illegible] and died 48 hours and 9 days post-operatively. In both cases at post-mortem gangr[illegible] ous bowel due to a small bowel volvulus was found. The other cases were all trea[illegible] by gastric suction and intravenous fluids for a variable time, usually for several d[illegible] (1 case for as long as 14 days) before it was decided to re-operate.

In the later group there were 17 cases and 4 of these died. Four cases develop[illegible] sudden severe colic and vomiting; this led to almost immediate laparotomy and [illegible] did very well. The remaining cases developed more insidiously and were treated [illegible]

PLATE XVIII

*A supine X-ray of the abdomen of a young man with a two hour history of internal strangulation. There is an almost total absence of gas, yet at operation 4 feet of strangulated small bowel was found.*

rst by gastric suction. This treatment was continued for periods up to 14 days; peration then became imperative. Four of these died, one was not operated on and ne others died within 24 hours of the second operation. Fig. 4 shows all the cases of ost-operative obstruction arranged in columns according to the length of treatment y gastric suction before the second operation, the height of each column representing he average length of stay in the hospital after the second operation and the shaded reas representing the deaths. It is clearly shown that the morbidity and mortality ncrease with delay in relieving the obstruction.

FIG. 4. *Shows how the average stay in hospital after the second operation increased with the time spent on treatment with gastric suction before operation in cases of poor operative obstruction. The figures at the end of each column represent the number of cases. The black areas denote deaths.*

The management of these patients is difficult, and the decision to re-operate a serious one. It would appear that, especially when obstruction develops several days after the primary surgery, early re-operation would be a wise course. Continued gastric aspiration only seems to disguise an established obstruction and the patient will deteriorate once any initial dehydration has been corrected.

### LARGE BOWEL OBSTRUCTION

Acute large bowel obstruction occurred in 45 cases and there were 21 deaths (47 per cent). This high mortality may be spurious, because some other cases may have been relieved by simple measures and then have had an elective operation with a [illegible] prognosis. Carcinoma of the colon was the commonest cause. There were 32 cases, 19 of these being situated in the sigmoid, 10 in the transverse, and 3 in the [illegible] colon. There were 6 cases of volvulus, 2 of diverticulitis, and 1 each of carcino[illegible] the bladder, simple stricture and stricture of a colostomy. In addition there w[illegible] cases who both died with gross abdominal distension and faecal impaction follo[illegible] the prolonged use of morphia.

Many of these patients were elderly and had other serious concomitant dis[illegible] several of the elderly refused treatment when that meant a colostomy. Many [illegible] carcinoma cases were very ill on admission with anaemia and dehydration from [illegible] standing obstruction. Fifteen (50 per cent) of the obstructions due to carcinoma [illegible] one death followed a later resection but all the others died as a direct result of the[illegible] struction, complicated often by associated cardio-respiratory disease, or metasta[illegible]

A few of the early cases of neoplastic obstruction were treated by primary rese[illegible] but most by laparotomy and the establishnemt of a colostomy. It would seem [illegible] even this is too large a procedure in the very poor risk case and a blind caecos[illegible] might be used more often. This could be followed by a more satisfactory colos[illegible] or a resection as soon as the patient is fit.

Sigmoin volvulus in 1 case required resection and this patient survived. Two [illegible] cases died, one as the result of a further volvulus and the other who was very [illegible] admission was treated by rectal decompression but died shortly afterwards. The [illegible] 4 cases were all treated by laparotomy, untwisting the volvulus and the passage [illegible] rectal tube.

### VASCULAR CAUSES OF OBSTRUCTION

This is an uncommon but important cause of obstruction because of the un[illegible] factory methods of treatment and the poor prognosis. There were only 8 cases [illegible] of these died. According to the Registrar General's Report (1955) this is an incre[illegible] cause of death, the crude death rate per million having increased fourfold between [illegible] and 1955. Three patients had mesenteric emboli and three mesenteric arterial th[illegible] bosis; these cases were all explored and infarction of all the bowel supplied by [illegible] superior mesenteric artery was found. None of these cases had the vessel expl[illegible] and they all died shortly after the operation.

In the early stages after mesenteric embolus there are very few signs to be f[illegible] in the abdomen and the patient remains surprisingly well for several hours. [illegible] diagnosis is therefore difficult, but should be considered in any patient with auric[illegible] fibrillation who has an attack of severe abdominal pain. Early diagnosis should [illegible] to exploration of the superior mesenteric artery for successful embolectomies [illegible] now been reported (Shaw and Rutledge, 1957).

The 2 cases that survived were probably cases of mesenteric venous thromb[illegible] as both had a previous history of venous thrombosis in other veins. These 2 cases [illegible] treated by intravenous heparin and antibiotics, and if the non-operative diagnos[illegible] venous thrombosis could be made with confidence then this is probably the [illegible] method of treatment.

### CONCLUSIONS

A consecutive series of 440 cases of acute small and large bowel obstruction [illegible] been presented. Seventy-one (15·5 per cent) cases died, some of severe associ[illegible] disease, but many as a direct result of the obstruction. The factor still chiefly resp[illegible] ible for the high mortality is delay in diagnosis.

The mortality rate has improved from 26 per cent given by Vick for the pe[illegible] 1925–1930, but the improvement is not in proportion throughout the series. [illegible] prognosis has improved most in the cases of internal strangulation, for the morta[illegible] rate has improved from 34 per cent in 1932 to 4 per cent in this series. Strangul[illegible] femoral hernia has also shown a marked improvement from 16 per cent to 6 per c[illegible]

but obstruction from non-strangulating adhesions has only changed from 32 per cent to 20 per cent. The mortality of strangulated inguinal hernia and vascular obstruction remains the same.

The improvement in the cases of internal strangulation is in part at least due to earlier admission to hospital. Most of Vick's cases were admitted on the second day but in this series two thirds of the cases reached hospital on the first day. The average length of history in the cases without strangulation has not changed to the same extent and there has consequently been less improvement. Similarly there has been an increase in the proportion of femoral herniae reaching hospital on the first day, but the length of history of the inguinal herniae remains about the same. The prognosis has improved in the former but remains constant in the latter.

Since 1930 great advances in fluid and electrolyte therapy, blood and plasma transfusion, antibiotics and anaesthesia have taken place. These have all contributed greatly to the improved prognosis, but their value lies in the management of the late case admitted after several days of vomiting, the case who is dehydrated, distended, and in urgent need of resection. These aids are rarely needed in the early case.

The most important cause of the high mortality in intestinal obstruction remains delay in operating. The delay is brought about firstly by difficulty in diagnosis, but it is also sometimes prolonged by the use of the gastric suction tube for periods longer than are required for rehydration.

Vick in 1932 said, "If we accept the contention that by early diagnosis and treatment these people may be saved from miserable death then these mortality rates are too high." This statement remains true today, for many of the deaths in this series could have been prevented by an earlier diagnosis.

### REFERENCES

*British Surgical Practice*, 1948. London, Butterworth and Co.

Registrar General's Report, 1955.

Shaw, R. S. and Rutledge, R. H. 1957. *N. England J. Med.*, **257**, 595.

Smith, G. A., Perry, J. F., and Yonehiro, E. G., 1955. *Surg. Gynae. Obst.*, **100**, 651.

## Figures and Tables

**Fig. 1. f1:**
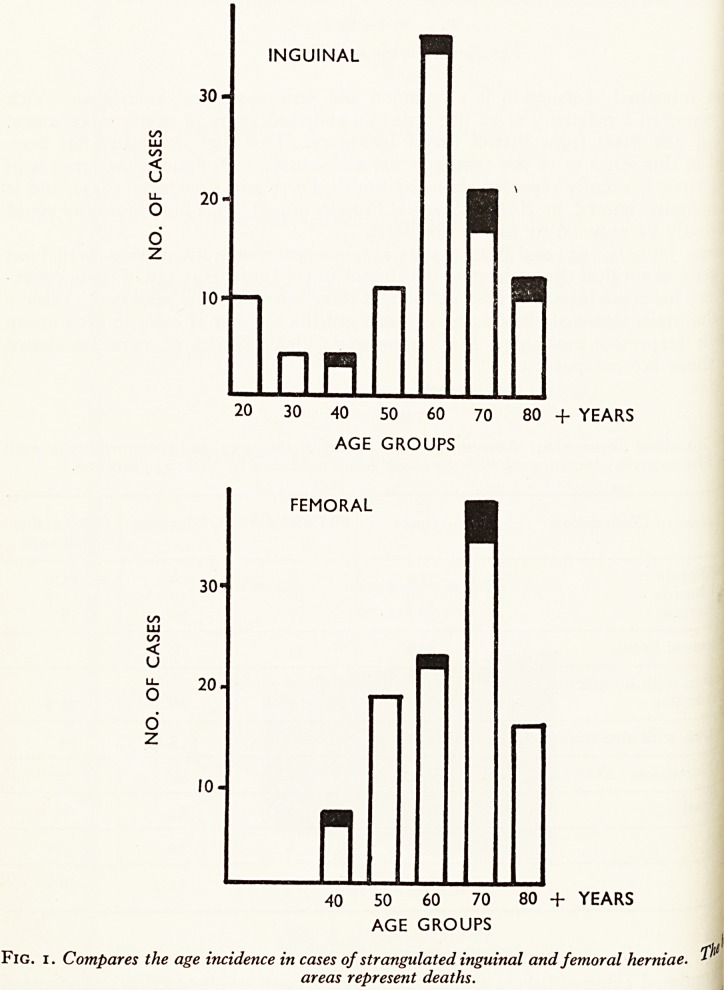


**Fig. 2. f2:**
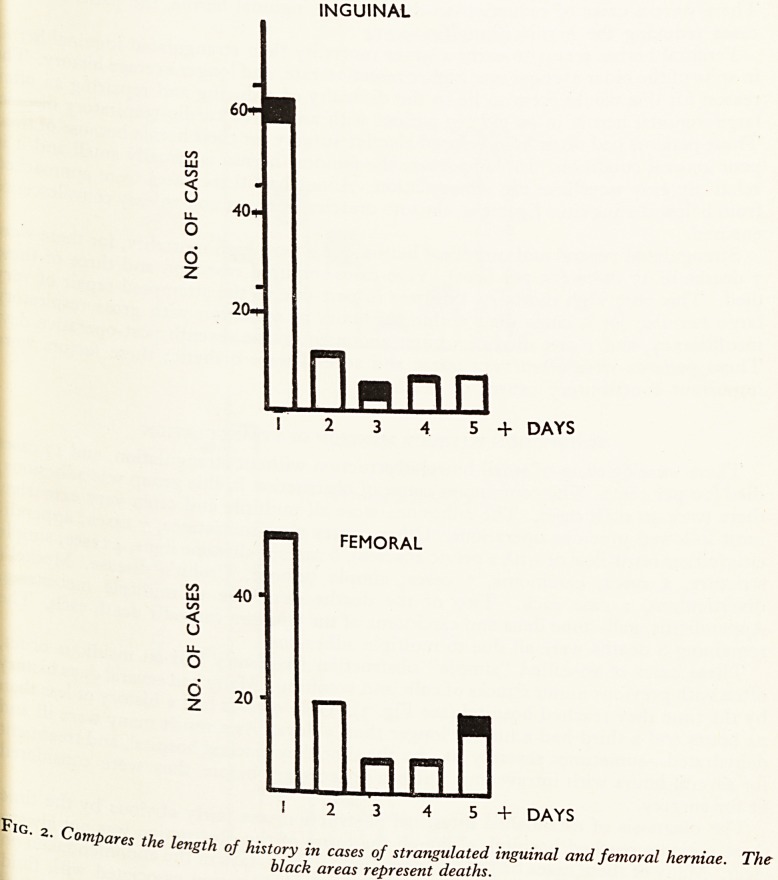


**Fig. 3. f3:**
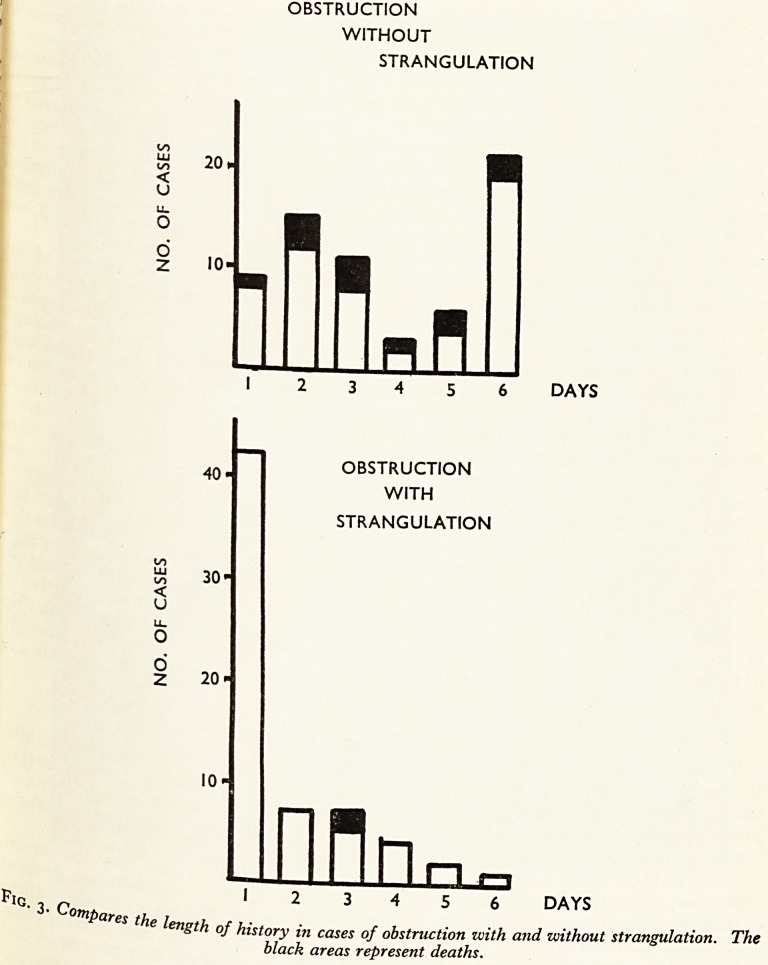


**Figure f4:**
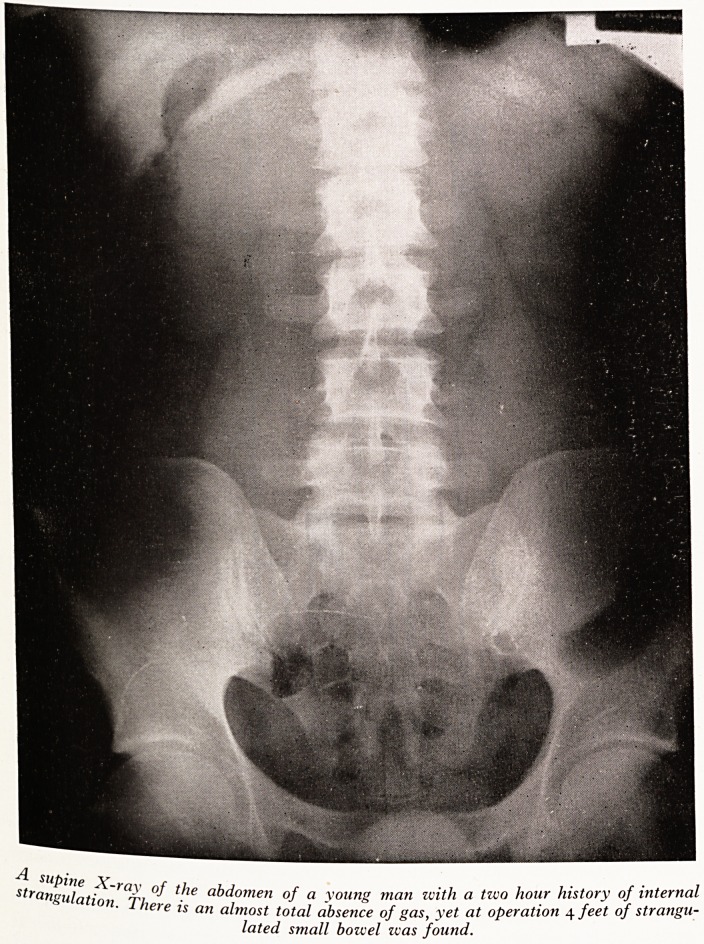


**Fig. 4. f5:**